# Prevalence and predictors of generalized anxiety disorder in women during the postpartum period

**DOI:** 10.1192/j.eurpsy.2025.2399

**Published:** 2025-08-26

**Authors:** S. Cipolla, P. Catapano, C. Toni, M. Di Vincenzo, C. Brandi, M. Luciano, G. Sampogna, A. Fiorillo

**Affiliations:** 1 Department of Psychiatry, University of Campania “L. Vanvitelli”, Naples, Italy

## Abstract

**Introduction:**

Up to 10% of women experience severe anxiety symptoms during pregnancy and the postpartum period, which are often underdiagnosed and undertreated, leading to negative outcomes for both mother and child.

**Objectives:**

This observational study aims to assess: 1) the prevalence of Generalized Anxiety Disorder (GAD) in the postpartum period, and 2) identify its predictors.

**Methods:**

All women attending the Gynecology and Obstetrics Department at “L. Vanvitelli” University Hospital were invited to participate in the study. Women who provided consent were assessed within three days after delivery using a specifically designed form for sociodemographic and clinical data collection, the Labor and Delivery Questionnaire (LDQ) for obstetric and gynecological information, and the Italian versions of the following assessment tools: Edinburgh Postnatal Depression Scale (EPDS) and the Generalized Anxiety Disorder 7-item scale (GAD-7). A GAD-7 score of ≥10 was used as the cutoff for moderate to severe Generalized Anxiety Disorder.

**Results:**

A sample of 110 women with a mean age of 30.74 (±5.67) years, predominantly Caucasian (91.8%), was recruited. Of these, 18.8% (n = 20) had GAD-7 scores indicating a diagnosis of Generalized Anxiety Disorder. Compared to women without GAD, those with GAD were significantly more likely to be unemployed or face difficulty finding work (p < 0.05), have a family history of anxiety disorders (p < 0.05), have other children (p < 0.05), experience conflicts with their parents (p < 0.001), and score higher on the EPDS (p < 0.001). Logistic regression analysis showed a higher likelihood of having GAD among younger women (OR: – 0.029; p < 0.05), those with a positive family history of anxiety disorders (OR: 0.63; p < 0.05), and those with higher EPDS scores (OR: 0.044; p < 0.001).

**Conclusions:**

The study highlights that age, employment status, and a family history of anxiety disorders may be significant predictors of GAD in peripartum period. Further studies with larger samples are necessary to confirm these findings; however, collaboration between psychiatrists, gynecologists, and obstetricians is recommended to identify women at risk of developing GAD early on, facilitating timely and appropriate diagnosis and treatment, and reducing the risks to the mental health of both mother and child.

**Disclosure of Interest:**

None Declared

